# Sodium channel biophysics, late sodium current and genetic arrhythmic syndromes

**DOI:** 10.1007/s00424-017-1959-1

**Published:** 2017-03-06

**Authors:** Karan R. Chadda, Kamalan Jeevaratnam, Ming Lei, Christopher L.-H. Huang

**Affiliations:** 1 Faculty of Health and Medical Sciences, University of Surrey, VSM Building, Guildford, GU2 7AL UK; 2 Physiological Laboratory, University of Cambridge, Downing Street, Cambridge, CB2 3EG UK; 3 School of Medicine, Perdana University–Royal College of Surgeons Ireland, 43400 Serdang, Selangor Darul Ehsan Malaysia; 4 Department of Pharmacology, University of Oxford, Oxford, OX1 3QT UK; 5 Department of Biochemistry, University of Cambridge, Hopkins Building, Cambridge, CB2 1QW UK

**Keywords:** Sodium channel activation, Sodium channel inactivation, Late sodium current, Paramyotonia congenita, Cardiac arrhythmic syndromes

## Abstract

Arrhythmias arise from breakdown of orderly action potential (AP) activation, propagation and recovery driven by interactive opening and closing of successive voltage-gated ion channels, in which one or more Na^+^ current components play critical parts. Early peak, Na^+^ currents (*I*_Na_) reflecting channel *activation* drive the AP upstroke central to cellular activation and its propagation. Sustained late Na^+^ currents (*I*_Na-L_) include contributions from a component with a delayed *inactivation* timecourse influencing AP duration (APD) and refractoriness, potentially causing pro-arrhythmic phenotypes. The magnitude of *I*_Na-L_ can be analysed through overlaps or otherwise in the overall voltage dependences of the steady-state properties and kinetics of activation and inactivation of the Na^+^ conductance. This was useful in analysing repetitive firing associated with paramyotonia congenita in skeletal muscle. Similarly, genetic cardiac Na^+^ channel abnormalities increasing *I*_Na-L_ are implicated in triggering phenomena of automaticity, early and delayed afterdepolarisations and arrhythmic substrate. This review illustrates a wide range of situations that may accentuate *I*_Na-L_. These include (1) overlaps between steady-state activation and inactivation increasing *window current*, (2) kinetic deficiencies in Na^+^ channel inactivation leading to *bursting phenomena* associated with repetitive channel openings and (3) *non-equilibrium gating* processes causing channel re-opening due to more rapid recoveries from inactivation. All these biophysical possibilities were identified in a selection of abnormal human *SCN5A* genotypes. The latter presented as a broad range of clinical arrhythmic phenotypes, for which effective therapeutic intervention would require specific identification and targeting of the diverse electrophysiological abnormalities underlying their increased *I*_Na-L_.

## Introduction

Arrhythmias follow disruption of the normal interacting succession of ion channel activation and inactivation that produces the transmembrane currents underlying the propagated action potential (AP) [10, 23, 80]. In skeletal muscle, these manifest as a range of syndromes associated with repetitive action potential firing associated with a number of ion channel abnormalities. Where involving the heart, ventricular arrhythmia potentially results in sudden cardiac death (SCD), which accounts for ∼4 to 5 million deaths per year worldwide [17]. Cardiac ischaemia accounts for most cases of arrhythmia [9], but ∼10–20% of arrhythmic deaths may result from ion channelopathy [40]. These could affect ion channels carrying Na^+^, *I*_Na_, and Ca^2+^ depolarizing currents, *I*_Ca_, and a number of, *I*_to_, *I*_Kr_, *I*_Ks_ and *I*_K1_, K^+^ channels contributing repolarizing current [57, 70]. The depolarisation-activated *I*_Ca_ induces Ca^2+^ release from intracellular sarcoplasmic reticular Ca^2+^ stores which triggers mechanical activity. The detailed AP timecourse in different cardiac regions or animal species is determined by their corresponding patterns of ion channel expression [71]. Once generated, local circuit currents driven by the inward flux of Na^+^ propagate APs to hitherto quiescent myocardial regions through gap junctions between successive cells. The result is a wave of membrane depolarisation followed by refractoriness [39].

The Na^+^ channel is central to this excitation process in view of its strategic role in initiation of the cardiac AP. The Na^+^ current, *I*_Na_, may comprise a mixture of currents with different kinetics. These might arise from modulations in the principal Na_v_ species or distinct channel subpopulations [67, 68]. Functional alterations in the biophysical properties of the Na^+^ channel thus lead to a range of arrhythmic conditions. An important group of these is the result of sustained inward Na^+^ current. The repetitive firing observed in skeletal muscle fibres in patients with *paramyotonia congenita* arises from an incomplete voltage-dependent Na^+^ channel inactivation thus leaving some channels in a conductive state [45]. This results from mutations in the skeletal muscle Na_v_1.4 channel [22, 44, 69] that produce positive shifts in the half maximal voltage of its steady-state inactivation function (*V*_inact_). Alternatively, negative shifts in the voltage dependence of Na^+^ channel activation (*V*_act_) permit channel activation with smaller depolarisations. Either situation potentially results in sustained inward current that follows excitation that can cause recurrent channel re-activation [12, 28, 29].

In cardiac muscle, sustained inward Na^+^ currents also known as late Na^+^ currents (*I*_Na-L_) occur under physiological conditions during the cardiac AP. These currents nevertheless have conductance, mean open time and selectivity properties identical to the remaining Na_v_1.5 current [25, 37]. It remains possible to consider both early Na^+^ currents and potentially arrhythmogenic *I*_Na-L_ in terms of the overall activation and inactivation characteristics that they produce in cardiac myocytes. A comparison with clinical findings will demonstrate that cardiac muscle shows a wider range of possible variations in such characteristics than the straightforward shifts in steady-state activation and inactivation reported so far in skeletal muscle. These findings have implications for therapeutic intervention.

### Na^+^ channel activation and inactivation

The cardiac Na^+^ channel multi-unit protein comprises principal Na_v_1.5, α- and associated auxiliary β-subunits. The α-subunit consists of four homologous domains (I–IV) each containing six transmembrane segments (S1–S6) [8], and it suffices to mediate ion selectivity, and the voltage-gated activation and inactivation properties of the channel [62]. Voltage-gated Na_v_1.5 *activation* depends on transitions in the S4 segment whose positively charged amino acids at every third position likely subserve a voltage-sensing function [13]. Membrane depolarisation moves the S4 segment relative to other channel segments so that the voltage-sensing domain, formed by the S1–S4 block, rotates. This permits Na^+^ influx through the pore-forming component made up of the S5 and S6 segments and the re-entrant P loop [21, 50]. This early *I*_Na_ drives the rapid AP upstroke resulting in further channel activation. Hodgkin and Huxley (1952) had first described such activation in terms of three *m* particles undergoing a voltage-dependent, first-order transition from an inactive to active state, giving channel openings of higher-order kinetics [32]. The maximum attainable Na^+^ current would depend upon the number of available channels, and be compromised in situations of Na_v_1.5 insufficiency, as in Brugada syndrome (BrS) [42].

Na_v_1.5 *inactivation* terminates the inward *I*_Na_ permitting membrane repolarisation also driven by other, outward, ionic currents. Na_v_1.5 inactivation involves two, fast and slow, kinetic components. Fast inactivation occurs within milliseconds and results from the cytoplasmic III–IV linker occluding the pore [38]. This may involve an isoleucine-phenylalanine-methionine (IFM) motif, which is a hydrophobic triplet in the III–IV linker that may act as a ‘latch’ keeping the fast inactivation gate shut [38]. Docking sites for this inactivation gate likely include the S6 segment in domain IV and the S4–5 loops in domains III and IV [8]. Hodgkin and Huxley correspondingly described a parallel first-order, voltage-dependent *h*-inactivation process resulting in refractoriness with prolonged depolarisation and recovery from such refractoriness with repolarisation. Slow inactivation, subsequently reported in NaF-perfused *Loligo* axons [14, 63], may include structurally distinct components [83], likely involving conformational changes of the pore component of the α-subunit [81].

**Fig. 1:**
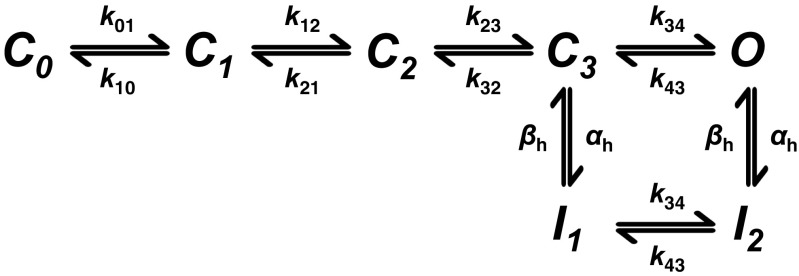
Sequence of Na^+^ channel states. State diagram representing transitions between closed (*C*), open (*O*) and inactivated (*I*) states of the Na^+^ channel incorporating ionic and gating current data, showing the voltage-dependent rate constants (*k*_mn_, *α*_*h*_ and *β*_*h*_) which determine the kinetics of transitions between states [81]

**Fig. 2:**
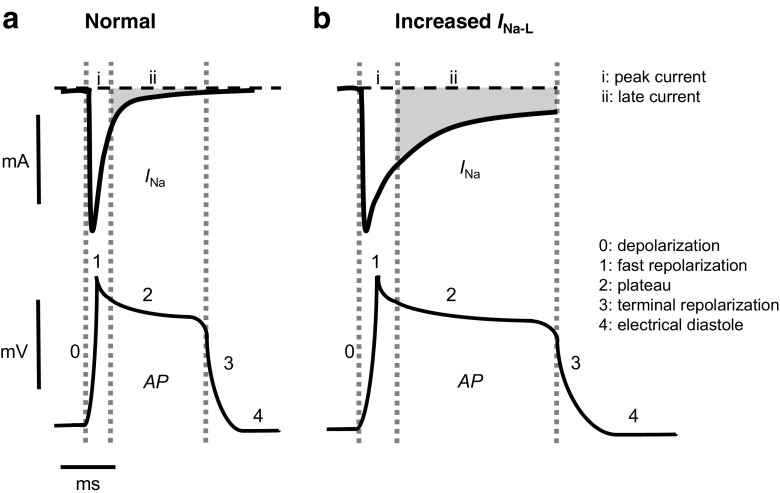
Relationships between peak and late Na^+^ currents and cardiac action potential timecourse. Comparisons of situations expected under conditions of normal (**a**) and prolonged action potential (AP) recovery timecourse (**b**). This illustrates the increase in amplitude and duration of late Na^+^ current (*I*_Na-L_) (*top panels*) in relationship to the timecourse of the successive phases (0–4) of the cardiac AP (*bottom panels*) under normal (**a**) and conditions associated with increased *I*_Na-L_ (**b**). Changes in *I*_Na-L_ magnitude are not drawn to scale

These processes have been organised in a reaction scheme in which a channel transitions through several closed (*C*_0_ to *C*_3_) resting states, then an open (*O*) state, with voltage-dependent rate constants *k*_mn_ intervening between any given pair of states *m* and *n*. The channel then transitions through inactivated (*I*_1_ and *I*_2_) states, followed by recovery from inactivation, similarly governed by voltage-dependent rate constants *α*_*h*_ and *β*_*h*_ (Fig. 1). The latter suggests resting, activated and inactivated states of the channel in which the channel is closed during resting and inactivated states, with separate processes mediating activation and inactivation. Channel opening with depolarisation is dependent not only upon the extent of activation but also upon the extent to which channels have transitioned into an inactivated state. These openings increase the membrane Na^+^ conductance, *g*_Na_, in turn permitting a peak *I*_Na_ or *I*_Na-L_ to take place down its net electrochemical driving force (*V*-*E*_Na_), contributing to the in vivo waveform of the cardiac AP (Fig. 2a). An early peak *I*_Na_ related to the activation process of the channel drives the upstroke of the AP and rapidly inactivates within a few milliseconds. The Na^+^ channel component underlying *I*_Na-L_ shows a diminished or slowed inactivation and a more negative (20 mV) voltage dependence in its activation properties than the remaining *I*_Na_ [68]. Early modelling predicted an *I*_Na-L_ of magnitude ∼1–2% of peak *I*_Na_ [48, 58]. Increases in *I*_Na-L_ thus influence AP duration and refractoriness. Currents arising from additional background *I*_bNa_ attributed to Na^+^-K^+^-ATPase and Na^+^-Ca^2+^ exchange-mediated leak currents are distinct from the voltage-dependent Na^+^ channel processes analysed here [7, 15, 31, 58].

### Graphical representation of Na^+^ channel activation and inactivation

**Fig. 3:**
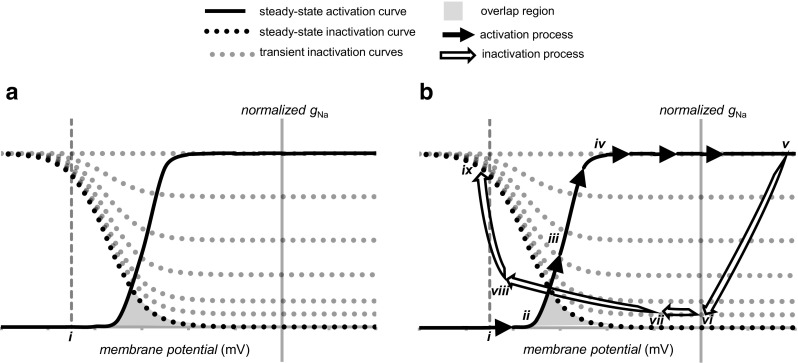
Curves illustrating the voltage dependence for Na^+^ channel activation and inactivation under steady-state and transient conditions. **a** Normalised steady-state activation and inactivation curves (after [51]) as well as a family of transient inactivation curves expected at successively greater intervals following the onset of a large voltage step (**a**) from the resting potential (*i*). Na^+^ conductance (*g*_Na_) is normalised to peak *g*_Na_ obtained in response to a depolarizing voltage step of sufficient magnitude to elicit maximum peak Na^+^ conductance. **b** Superimposed upon these activation curves is an illustration of the trajectory of Na^+^ current activation (*continuous line*, *arrowed*) from the resting potential (*i*) through voltages along the increasing (*ii*)–(*iv*) and plateau regions (*iv*) and (*v*) of the activation curve. This is followed by the trajectory of Na^+^ current inactivation from the action potential peak (*v*) through phase 1 rapid repolarisation ((*v*)–(*vi*)), the phase 2 plateau ((*v*i)–(*vii*)), phase 3 repolarisation ((*vii*)–(*viii*)) and restoration of electrical diastole ((*viii*)–(*ix*)), over which the Na^+^ channel recovers from refractoriness. The overlap between the activation and inactivation curves is shaded *grey* to illustrate conditions under which a physiological window current would be expected

Figure 3 illustrates graphical representations of the consequences of these activation and inactivation processes and their possible interaction, which predicts the resulting *I*_Na_. First, the steady-state activation curve illustrates the potential increase in Na^+^ conductance and therefore of *I*_Na_ as a function of the membrane potential *V*. Each individual *I*_Na_ component would reflect a conductance contribution *g*_Na_ described by a Boltzmann distribution between two, open and closed, states whose energies that vary linearly with *V*. This would predict a sigmoid relationship between *g*_Na_ and *V*. In the equation,

$$ {g}_{\mathrm{Na}}= g{{}_{\mathrm{Na}}}^{\ast }/\left\{1+ \exp \left[-\left( V-{V}_{\mathrm{act}}\right)/{k}_{\mathrm{act}}\right]\right\}, $$

term *g*_Na_^*^ is the maximum value of *g*_Na_ and *V*_act_ is the voltage at its half-maximum value. The term *k*_act_ is the slope factor for activation, which characterises the voltage sensitivity of the component channel in terms of the valency, *z*, of the charge transfer involved in its transitions between the open and closed states, through the equation *k*_act_ = *RT*/(*zF*), where *R* = gas constant, *T* = temperature and *F* = Faraday’s constant. Either increasing the value of z or reducing the value of *k*_act_ will increase the steepness of the activation-voltage relationship.

Second, steady-state inactivation curves normalised to the interval [0,1] give an indication of the fraction of activatable channels mediating each Na^+^ conductance component, *h*, as limited by the degree of inactivation, each with its own inactivation slope factor *k*_inact_,

$$ h=1/\left\{1+ \exp \left[-\left( V-{V}_{\mathrm{inact}}\right)/{k}_{\mathrm{inact}}\right]\right\}. $$

Third, in addition to their steady-state properties, the activation and inactivation processes show distinct kinetics. Inactivation kinetics is typically slower than activation kinetics and extends over timescales comparable to the recovery phase of the AP. A family of inactivation curves rather than a single inactivation curve could be used to represent the time evolution of the inactivation process as might occur following imposition of a given voltage step. For any given component, the simplest two-state model might assume activatable and inactivated states of energies *E*_1_(*V*) and *E*_2_(*V*), respectively, linearly dependent on the voltage *V* through a coefficient dependent on their effective position in the membrane field. It could then incorporate forward, *α*, and backward, *β*, rate constants determined by the energy of the barrier *E* ^*^(*V*) given by *α*(*V*) = *A* exp{[*E*_1_(*V*) − *E* ^*^(*V*)]/*kT*} and *β*(*V*) = *A* exp{[*E*_2_(*V*) − *E* ^*^(*V*)]/*kT*}, where *k* in this instance represents the Boltzmann constant and *A* the Arrhenius constant [1].

### The relationship between Na^+^ channel activation and inactivation curves and the late Na^+^ current, *I*_Na-L_

The relative contributions of activation and inactivation properties can be assessed from plots of their respective overall dependencies upon voltage. These are shown in functions illustrated using established experimental values in Fig. 3a [51]. An imposed voltage step would produce a rapid activation of *g*_Na_, whose value would fall close to the corresponding ordinate of the activation curve. Thus, there will initially be little evidence of the slower inactivation process, but this will subsequently cause *g*_Na_ to decline. This time evolution of inactivation is represented by the successive dotted transient inactivation curves that progressively approach the steady-state inactivation curve, which would predict full inactivation at depolarised voltages. Alternatively, a persistent conductance would result from an incomplete inactivation either at times when the relevant decay is incomplete or in the event of an incomplete steady-state inactivation at the voltage in question. Thus, the effective *g*_Na_ at any given time following a particular voltage step is effectively the ordinate in the activation multiplied by corresponding ordinate in the inactivation function.

Figure 3b reconstructs both the *g*_Na_ activation (full arrows) and inactivation variables (open arrows) through the timecourse of the cardiac AP. The charging of an initially quiescent membrane at the resting potential (i) by local circuit currents through the passive cable formed by intervening cardiac myocytes from previously excited membrane regions produces an activation locus ((i)–(ii)) to the foot of the activation-voltage relationship. The resulting Na^+^ channel opening initiates a regenerative cycle of depolarisation and further channel opening producing the steep rise of *g*_Na_ along the activation curve from ((ii)–(iii)) to maximum channel activation along ((iv)–(v)), thereby completing phase 0 of the cardiac AP. The inactivation locus is then followed through phase 1 fast early repolarisation ((v)–(vi)), during which there is a rapid Na^+^ channel inactivation. This is succeeded by the phase 2 plateau ((vi)–(vii)), during which an incomplete development of inactivation leaves a finite *I*_Na-L_. Locus ((vii)–(viii)) traces phase 3 terminal repolarisation and a return to electrophysiological diastole at the resting potential ((viii)–(ix)). Recovery from inactivation is favoured at membrane potentials near to the resting potential and this then permits re-excitation.

### Importance of late Na^+^ current, *I*_Na-L_

Figure 2a illustrates a presence of *I*_Na-L_ during the AP plateau phase, whereas Fig. 2b illustrates circumstances of increased *I*_Na-L_. In addition to extending the plateau duration before AP recovery, an increased *I*_Na-L_ can lead to the development of various triggers and substrates for arrhythmogenesis. First, it can cause diastolic depolarisation phenomena, which trigger inappropriate APs in the sino-atrial node (SAN) and the potentially pacemaking atrioventricular node (AVN) and His-Purkinje cells. This can result in an abnormal automaticity reduced by inhibiting such *I*_Na-L_ [23, 76]. Second, enhanced *I*_Na-L_ can predispose to afterdepolarisations during or immediately following an AP [87]. Of these, early afterdepolarisations (EADs) occur during phase 2 or 3 of a prolonged AP [5, 23]. This then causes a regenerative L-type Ca^2+^ channel re-activation, whilst the membrane remains depolarised during a prolonged AP plateau phase. EADs have been observed both in genetic conditions such as long QT syndrome (LQTS) and acquired conditions such as cardiac failure [36]. Delayed afterdepolarisations (DADs) follow full repolarisation in cells with Ca^2+^ overload. These in turn predispose to depolarizing, transient inward currents. Although smaller than the peak *I*_Na_, *I*_Na-L_ has a 50–100-fold longer duration and thereby can increase cellular Na^+^ loading, in turn reducing the gradient for Ca^2+^ efflux through sodium-calcium exchange current [58]. DADs may underlie arrhythmias seen in some heart failure patients, patients with digitalis toxicity and patients with catecholaminergic polymorphic ventricular tachycardia [5].

**Fig. 4:**
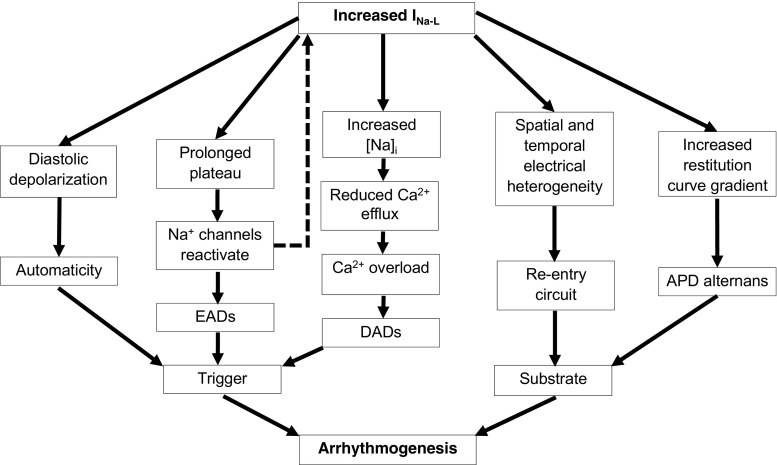
Simplified scheme illustrating mechanisms by which increased late sodium current can promote arrhythmogenesis

Third, the presence of *I*_Na-L_ also bears upon *re-entrant processes* re-exciting recovered regions, thus furnishing *arrhythmic substrate* perpetuating the initial arrhythmic event [3]. *I*_Na-L_ upregulation by *Anemonia sulcata* toxin (ATX-II) increased dispersion of repolarisation and refractoriness. This could lead to the torsades de pointes associated with LQTS [4]. Finally, *I*_Na-L_ also increases the slope of the AP duration (APD) restitution curve relating AP duration to the diastolic interval intervening between AP recovery and generation of the subsequent AP during regular stimulation at successively higher frequencies [59, 78]. A pathological *I*_Na-L_ upregulation therefore promotes arrhythmic triggers and substrates through a variety of pathways, summarised in Fig. 4.

### *I*_Na-L_ and altered g_Na_ activation

**Fig. 5:**
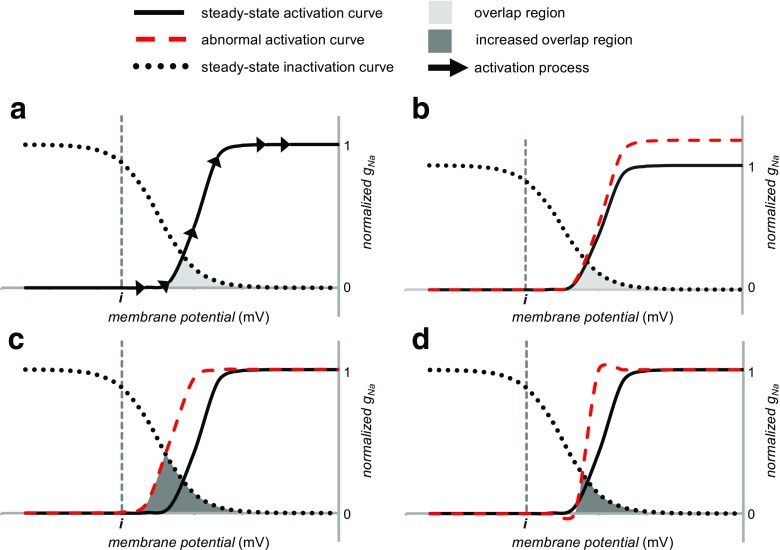
Changes in the activation curve that would increase a depolarizing *I*_Na_ (**a**). The normal activation curve and the activation trajectory following a depolarizing voltage step sufficient to cause maximum activation. This is compared with the steady-state inactivation curve. **b** Illustration of the consequences of an increase in maximum sodium conductance (*g*_max_) without a change in its voltage sensitivity (*k*_act_). An increase in maximum activation takes place with relatively little change in the slope or the position of the curve along the horizontal axis. This would simply increase the peak sodium conductance. **c** Shift of the activation curve along the negative direction decreasing *V*_act_, without altering its voltage sensitivity, *k*_act_. This increases the overlap between the activation and inactivation curves. This abnormality increases *I*_Na-L_ by increasing the window current. **d** Increases in the voltage sensitivity, corresponding to a decrease in *k*_act_. This increases the overlap between the activation and inactivation curves and similarly increases *I*_Na-L_ by increasing window current

Increased *I*_Na-L_ thus potentially constitutes a final common pathway explaining a wide range of pro-arrhythmic phenomena. However, a wide range of alterations in either or both of the biophysical properties of *g*_Na_ activation and inactivation (Fig. 5a) provide potential causative mechanisms for increased *I*_Na-L_. These range from alterations in maximum *g*_Na_ despite an otherwise unaltered activation-voltage plot, as could occur following an increased surface membrane Na^+^ channel expression (Fig. 5b). This situation would contrast with the consequences of some Brugada syndrome cases, which contain a Na_v_1.5 haploinsufficiency [51].

In the remaining cases, changes in either the steady-state or kinetic properties of activation and/or inactivation alters the interrelationship between these curves. This determines the size of *I*_Na-L_ whether in the form of a prolonged opening of Na^+^ channels or a re-opening of previously inactivated channels [58]. In situations in which there are overlap regions in the *steady-state* activation and inactivation functions, an experimental situation involving the imposition of voltage clamp steps would demonstrate a persistent steady-state equilibrium, *window current* [48, 54]. Similar overlaps could occur prior to achievement of a steady state. These would particularly arise from the kinetics of either activation or inactivation that would remain amenable to the graphical analysis adopted here.

Nevertheless, the relatively rapid kinetics of Na^+^ channel activation over timescales substantially preceding recovery processes related to *I*_Na-L_ permit its approximation by its steady-state activation curve properties. A number of activation curve variants could then potentially influence the existence or magnitude of *I*_Na-L_. Of these, negative shifts in the voltage dependence of activation quantified by negative changes in *V*_act_ have been reported with genetic modification in either the Na_v_1.5 α- or β-subunits [27]. Alternatively, this negative change could occur in situations shifting the electric field seen by the voltage sensor for activation with reductions in external or increases in internal Ca^2+^ or Mg^2+^ concentration (Fig. 5c) [52, 73, 75]. These would shift the foot of the activation voltage curve closer to the threshold voltage and increase the likelihood of re-activation phenomena. In addition, an increased steepness in the activation curve (Fig. 5d) quantified by a decrease in *k*_act_ and therefore an increase in the effective valency *z* could arise in mutations affecting the charge on the voltage sensor controlling Na^+^ channel gating.

### *I*_Na-L_ and altered g_Na_ inactivation

**Fig. 6:**
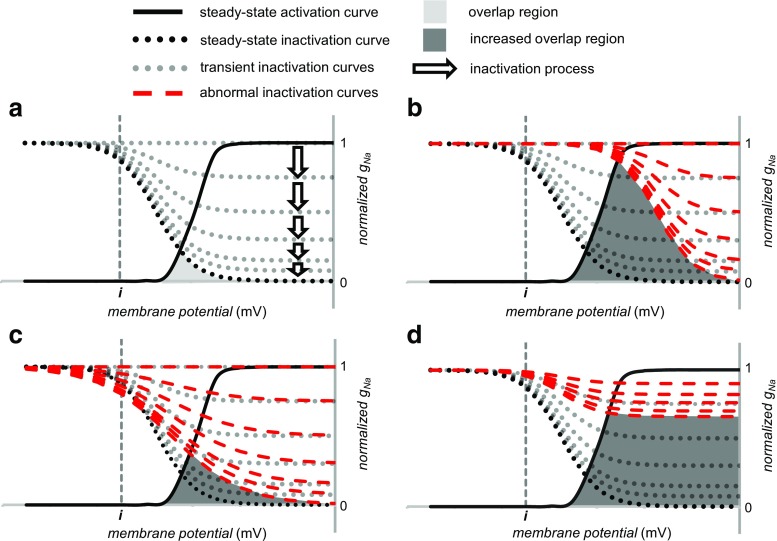
Changes in the inactivation curve that would increase a depolarizing *I*_Na_. **a** Family of normal inactivation curves obtained at different intervals (*arrows*) following imposition of a positive depolarizing voltage step producing an initial Na^+^ conductance activation along the activation curve. The *arrows* proceed vertically downward representing the transition to full steady-state inactivation as the membrane potential is clamped at a positive voltage. The length of the *arrow* denotes the rapidity of the process of inactivation is. **b** Positive shift of the inactivation curve along the voltage axis increasing *V*_inact_ without altering its voltage sensitivity *k*_inact_. This increases the overlap between activation and inactivation curves. This therefore increases *I*_Na-L_ by increasing window current. **c** Increase in *k*_inact_ decreasing the steepness of the inactivation curve. This also increases the overlap between the activation and inactivation curves, again increasing *I*_Na-L_ by increasing window current. **d** Slowing of inactivation kinetics. At any given time, the transient inactivation curves then assume higher values than shown by the normal transient inactivation curves. As a result, at any given time, fewer Na^+^ channels are inactivated, giving a higher sustained *I*_Na-L_ potentially producing bursting behaviour

Figure 6a illustrates both steady state and a family of curves (dotted lines) representing the kinetics of inactivation following a depolarizing voltage step, which explores the development of inactivation independently of its recovery. Both positive shifts, with increased *V*_inact_ (Fig. 6b) or a decreased steepness with increased *k*_inact_, of steady-state inactivation (Fig. 6c), could increase overlap between steady-state activation and inactivation, accentuating *I*_Na-L_ through an increased window current. In addition, alterations in inactivation *kinetics* could take place incidental to such shifts in steady-state inactivation or in a presence of otherwise normal steady-state properties. A slowing of the kinetics even in the absence of any steady-state abnormality (Fig. 6d) could result in a further mechanism of increasing *I*_Na-L_ manifested in *bursting phenomena*. Bursting reflects a *transient kinetic failure of Na* ^*+*^ *channel inactivation*. The resulting gating mode is associated with a small proportion of the channels alternating between the last closed available state and open state (Fig. 1), and these frequent openings could give rise to *I*_Na-L_ [18, 79].

**Fig. 7:**
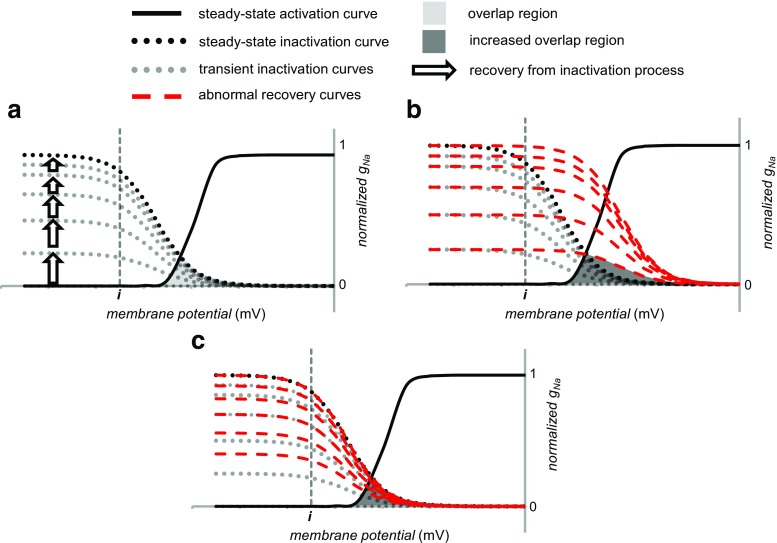
Changes in the recovery from inactivation following the repolarizing phase ending a voltage step. The membrane potential is returned to a negative voltage near the resting potential. **a** The normal recovery from inactivation processes represented by *upward pointing arrows* proceed through a succession of transient inactivation curves approaching the steady-state inactivation curve at full recovery. The length of the *arrow* denotes the rapidity of the process. **b** Positive shift in the voltage dependence of recovery from inactivation increases the overlap between activation and inactivation. **c** Faster kinetics for the recovery from inactivation also increases this overlap. Both situations increase *I*_Na-L_

Finally, voltage steps restoring the resting membrane potential drive Na^+^ channel recovery from inactivation over a refractory period (Fig. 7a). As in the case of inactivation, recovery kinetics can similarly follow altered (Fig. 7b) or take place in the presence of normal steady-state voltage dependencies of inactivation (Fig. 7c). This can increase *I*_Na-L_ by a *non-equilibrium gating process*. The latter could cause channel re-opening due to a *decreased recovery time from inactivation* during the terminal repolarisation phase of the AP. This re-opening could take place beyond the overlap region between steady-state activation and inactivation curves. Thus, rapid recovery of a small subpopulation of the channels from inactivation permits their immediate re-activation [19]. Even following full restoration of the resting membrane at the end of the step, the examples in Fig. 7b, c may show similarly altered recovery kinetics. Such altered kinetics might reflect shifted steady-state inactivation curves, which would corespondingly shift rate constants involving inactivation or recovery from inactivation along the voltage axis. They could also result from alterations in the rate constants themselves. Either would alter rates of recovery from inactivation at a given voltage.

### *I*_Na-L_ and clinical genetic arrhythmic conditions

The previous sections thus suggest a hypothesis invoking a convergence of a range of Na^+^ channel activation and inactivation abnormalities, all of which produce *I*_Na-L_. Table 1 summarises a selection of experimental studies using expression systems of a range of inherited clinical mutations in human *SCN5A*, all of which have been associated with increased *I*_Na-L_. It confirms that each mutation results in characteristics illustrating one or more of all the biophysical examples discussed above as illustrated by Figs. 5, 6 and 7. Conversely, the mutations in the table cover all these biophysical cases when taken together. Thus, an enhanced *I*_Na-L_ can result from a large variety of abnormalities in Na^+^ channel gating.

**Table 1:** Biophysical characterisation of a selection of SCN5A mutations related to an increased *I*_Na-L_ and clinical arrhythmic syndromes

Mutation	Increased maximum *g*_Na_	Negatively shifted *V*_act_	Decreased *k*_act_	Positively shifted *V*_inact_	Increased *k*_inact_	Slowed inactivation kinetics	Accelerated recovery from inactivation	Reference
Long QT syndrome								
p.R1644H	−	−	−	−	−	−	+	[49]
p.H1849R	+	−	−	+	−	+	−	[55]
R1626P	−	−	−	−	+	−	−	[65]
P1332L	−	+	−	−	−	−	−	[65]
M1652R	−	−	−	+	−	−	+	[65]
S216L	+	+	−	−	−	−	−	[61]
R568H	+	−	−	+	−	−	−	[61]
A572D	+	−	−	−	−	+	+	[61]
V411M	−	+	−	−	−	−	−	[33]
L409P/R558	−	−	−	+	−	−	+	[85]
P2006A	−	−	−	+	−	−	+	[72]
Complex arrhythmia and dilated cardiomyopathy								
R225P	+	+	+	−	+	−	−	[53]
R814W	−	+	+	−	−	−	−	[53]
Sudden infant death syndrome								
S1333Y	+	+	−	+	−	−	+	[34]
Multifocal ectopic Purkinje-related premature contractions								
p.R222Q	−	+	−	−	−	−	−	[43]
Atrial fibrillation								
p.H1849R	+	−	−	+	−	+	−	[55]
K1493R	−	−	−	+	−	+	−	[46]
Exercise-induced polymorphic arrhythmia								
p.I141V	−	+	−	−	−	−	−	[77]

In particular, Table 1 illustrates the above points in detail for long QT syndrome type 3 (LQTS3) using the relatively large number of examples from which selections can be made for analysis [33, 49, 55, 61, 65, 72, 85]. LQTS3 is one of a range of genetic (LQTS1–LQTS13) long QT syndromes characterised by prolonged QT intervals, reflecting increased ventricular APD and additional aberrant *T*-wave ECG signatures. They are all associated with a predisposition to normally self-terminating episodic polymorphic ventricular tachycardia (VT), torsades de pointes (TdP), with the potential to degenerate into ventricular fibrillation and/or SCD [2]. LQTS3 patients commonly exhibit bradycardia, and they show a greater risk of arrhythmia at lower heart rates, such as during rest and sleep [64]. The most common mechanism of LQTS3 pathophysiology is the disruption of the fast inactivation kinetics of the channel, enhancing *I*_Na-L_ [30]. However, the list of mutations in Table 1 suggests that each hypothetical alteration in activation and inactivation properties proposed above is associated with a particular case of LQTS3. The one exception concerns situations resulting in an increased steepness of the activation-voltage relationship and a consequent decrease in *k*_act_. Nevertheless, the latter phenotype was represented in the particular, R225P and R814W, mutations. These are associated with complex arrhythmias combined with dilated cardiomyopathy. They showed an increased voltage sensitivity of the Na^+^ channel. This took the form of a decrease in *k*_act_ without any change in the inactivation curve [53].

Conversely, a given *I*_Na-L_ phenotype could be associated with a wide range of clinical manifestations in different patients. In addition to LQTS3, these include sudden infant death syndrome (SIDS), multifocal ectopic Purkinje-related premature contractions (MEPPC) and atrial fibrillation (AF). SIDS is a significant cause of infant mortality, and arrhythmias are an important cause of SIDS, with inherited LQTS making up 9.5% of SIDS cases [6]. The S1333Y mutation in Table 1 associated with SIDS involves the domain III S4–S5 linker near the proposed docking site of regions implicated in channel inactivation. The mutant channel showed enhanced window and persistent inward currents, producing a LQTS3-like phenotype [34]. This mutation led to changes in both activation and inactivation processes that taken together would tend to increase *I*_Na-L_. Thus, the S1333Y mutation resulted in an increased maximum *g*_Na_, a negatively shifted *V*_act_ but a positively shifted *V*_inact_. There was also a more rapid recovery from inactivation. However, there does exist a A1330P mutation that has similar ionic channel effects and also causes SIDS, yet does not produce enhanced *I*_Na-L_ [34].

The autosomal dominant MEPPC syndrome is associated with an arrhythmia characterised by premature ventricular contractions that originate from ectopic foci along the fascicular-Purkinje system. This results in non-sustained VT and, depending on its severity, SCD [47]. A consistently observed window current may increase the excitability of the fascicular-Purkinje system [47]. Table 1 exemplifies one particular R222Q mutation, which clearly co-segregated with the MEPPC phenotype. This was uniformly present in three unrelated families, and dilated cardiomyopathy appeared as a secondary consequence. The triggered APs and premature ventricular contractions were attributed to altered voltage dependencies of Na_v_1.5 activation. They were absent at higher pacing frequencies, consistent with their disappearance during exercise [43]. Accordingly, as shown in Table 1, this MEPPC mutation resulted in a negatively shifted *V*_act_.

AF is an abnormal heart rhythm manifesting as rapid, irregular beating and palpitations; dyspnoea; dizziness; and chest pain [41, 55]. The gain-of-function K1493R mutation associated with AF showed a positively shifted *V*_inact_ that would increase the window current and reduce the excitation threshold and a slowed kinetics for inactivation [46]. However, mutations in genes other than *SCN5A* have also been associated with AF, including *SCN10A* and *SCN1B*. *SCN10A* encodes the voltage-gated Na_v_1.8 known to be highly expressed in intracardiac neurons [84]. Its role is not fully understood but mutations in it are associated with disease phenotypes. Thus, the A1073 variant increases risks of AF consistent with functional studies, demonstrating increased peak *I*_Na_, increased *I*_Na-L_ and prolonged fast inactivation [35]. Although peak Na_v_1.8 current is much smaller than peak Na_v_1.5 current, *I*_Na-L_ arising from Na_v_1.8 is 20–50 times higher than *I*_Na-L_ arising from Na_v_1.5. Therefore, Na_v_1.8-mediated *I*_Na-L_ could strongly influence APD [35]. Finally, a given *I*_Na-L_ phenotype could be associated with more than one clinical manifestation. Thus, a negatively shifted *V*_act_ arising from three different mutations, P1332L, p.R222Q and p.I141V, gave LQTS3, MEPPC and exercise-induced polymorphic arrhythmia, respectively [43, 65, 77].

### *I*_Na-L_ as a pharmacological target

Increased *I*_Na-L_ thus potentially triggers and provides substrate for arrhythmia under diverse circumstances. The analysis above would indicate that potential therapeutic candidates should target *I*_Na-L_ through their action on the steady-state voltage dependence of Na^+^ channel activation and/or inactivation and their kinetic properties. This would require such action to take directions and extents appropriate to minimizing *I*_Na-L_ in the particular condition concerned. Such an action could be further enhanced if the applied agent selectively acted upon *I*_Na-L_ as opposed to peak *I*_Na_.

This strategy is exemplified by recent explorations directed at paramyotonia congenita in skeletal muscle. For example, the anticonvulsant lamotrigine *negatively* shifted the inactivation *V*_1/2_, modifying inactivation kinetics and decreasing *I*_Na_, in a HEK293 expression system expressing Na_v_1.4 [56]. Rufinamide *positively* shifted the voltage dependence of *I*_Na_ activation in human Na_v_1.1 transiently expressed in *Xenopus* oocytes [24]. *Both* lamotrigine and rufinamide at concentrations appropriate for achieving clinical anticonvulsant activity reduced myotonia in isolated human and rat skeletal muscle [74]. This could provide a model for selective therapeutic modifications of activation and inactivation in the cardiac Na_v_1.5 channel. These could be targeted at patients with particular specific genetic mutations if their underlying electrophysiological abnormalities could be characterised in order to predict their response to these drugs. The latter in turn could prompt clinical trials to assess the efficacy of the resulting *I*_Na-L_ inhibitors on the arrhythmic variants concerned.

Studies investigating the precise effects of *I*_Na-L_ inhibitors on the biophysical properties of the Na^+^ channel could lead to therapy targeted at cardiac arrhythmias associated with increased *I*_Na-L_. LQTS patients who responded well to mexiletine carried mutations resulting in a negative shift in *V*_act_, whereas patients whose QT intervals were not modified in response to mexiletine did not have a negative shift in *V*_act_ [65]. This has implications in that it may be necessary to target the specific biophysical alteration in Na^+^ channel function to effectively inhibit the pro-arrhythmic effects of *I*_Na-L_. This would require further investigations of its biophysical actions on mutant Na_v_1.5. However, mexiletine did not affect voltage-dependent activation but negatively shifted steady-state fast and slow inactivation and markedly prolonged recovery from inactivation of Na_v_1.5. These actions culminated in a use-dependent *I*_Na_ block in expressed WT Na^+^ channels [86]. Nevertheless, mexiletine rescued negatively shifted steady-state activation voltage dependencies in *SCN7A*-L858F-mutated channels [20].

Recent reports have shown that ranolazine was 9 to 45 times more selective in inhibiting *I*_Na-L_ than peak *I*_Na_ in isolated canine ventricular myocytes [82]. It decreased QTc interval in a group of eight LQTS3 patients carrying *SCN5A*-D1790G. The blocking effect of ranolazine on *I*_Na-L_ was recapitulated in a TSA201 expression system [16]. Meta-analysis showed that ranolazine significantly reduced incidences of AF relative to control groups in various clinical settings [26, 66]. The trial compound GS-458967 similarly appeared to inhibit *I*_Na-L_ in preference to peak *I*_Na_ particularly in the atria as opposed to the ventricles [11]. The further agent vernakalant (RSD1235) did not show selectivity for *I*_Na-L_ over peak *I*_Na_ [60].

## Conclusions

Sodium currents (*I*_Na_) are strategic to cardiac excitation and are mediated by one or more Na^+^ channel components, reflecting different states in the cardiac Na_v_1.5 or Na^+^ channel species that together culminate in peak *I*_Na_ and late *I*_Na-L_ components. Accordingly, inherited Na_v_1.5 abnormalities can disrupt AP generation, propagation and recovery to cause arrhythmia. In particular, an increased *I*_Na-L_ leads to a variety of arrhythmic conditions and can develop from various biophysical alterations in the overall activation and inactivation gating properties of Na_v_1.5. Firstly, an increased overlap between the steady-state activation and inactivation functions can increase window current. Secondly, a transient kinetic failure of Na^+^ channel inactivation can lead to bursting phenomena, characterised by frequent channel openings. Finally, a *non-equilibrium gating* process can cause channel re-opening due to a decreased recovery time from inactivation. Available clinical evidence from different pro-arrhythmic Na_v_1.5 mutations can be used to illustrate each of the wide range of possible mechanisms. These various mechanisms for increasing *I*_Na-L_ could provide a useful basis for the selection of therapeutic agents for patients with the disease phenotype. Selectively targeting the specific biophysical change underlying the increased *I*_Na-L_ could improve efficacy and allow mutation-specific therapy. This would require preclinical characterisation of how the drug compounds affect the biophysical properties of the *I*_Na-L_. In turn, specifically tailored drug intervention in patients with any given genetic mutation would require characterisation of their underlying electrophysiological abnormalities in order to predict their response to these drugs.

## Abbreviations

*A*: Arrhenius constant
AF: Atrial fibrillation
AP: Action potential
APD: Action potential duration
ATX-II: *Anemonia sulcata* toxin
AVN: Atrioventricular node
BrS: Brugada syndrome
DAD: Delayed afterdepolarisation
EAD: Early afterdepolarisation
ECG: Electrocardiographic
*F*: Faraday’s constant
*g*_Na_: Sodium conductance
*g*_Na_^*^: Maximum value of sodium conductance
*I*_Ca_: Calcium current
IFM: Isoleucine-phenylalanine-methionine
*I*_K1_: Inward rectifying K^+^ current
*I*_Kr_: Rapid delayed rectifier K^+^ current
*I*_Ks_: Slowly activating delayed rectifier K^+^ current
*I*_Na_: Sodium current
*I*_Na-L_: Late sodium current
*I*_to_: Transient outward K^+^ current
*k*: Boltzmann constant
*k*_act_: Slope factor for activation
*k*_inact_: Slope factor for inactivation
*k*_mn_: Voltage-dependent rate constants
LQTS3: Long QT syndrome type 3
MEPPC: Multifocal ectopic Purkinje-related premature contractions
*R*: Gas constant
SAN: Sino-atrial node
SCD: Sudden cardiac death
SIDS: Sudden infant death syndrome
*T*: Temperature
TdP: Torsades de pointes
*V*: Membrane potential
*V*_act_: Voltage at half-maximum activation
*V*_inact_: Voltage at half-maximum inactivation
VT: Ventricular tachycardia
*z*: Valency
*α*_*h*_: Time constant for transition into inactivation
*β*_*h*_: Time constant for recovery from inactivation
